# HER2-Positive Metaplastic Spindle Cell Carcinoma Associated with Synchronous Bilateral Apocrine Carcinoma of the Breast

**DOI:** 10.1155/2014/310829

**Published:** 2014-09-18

**Authors:** Katsumi Kito, Toshiharu Maeda, Keiko Ninomiya, Atsuro Sugita, Teiri Sagawa, Kinya Matsuoka, Kousei Kinoshita, Naoki Hyodo, Nagisa Morita, Keizo Furuya

**Affiliations:** ^1^Department of Pathology, Ehime Prefectural Central Hospital, 83 Kasuga-cho, Matsuyama, Ehime 790-0024, Japan; ^2^Department of Surgery, Ehime Prefectural Central Hospital, Matsuyama 790-0024, Japan

## Abstract

Apocrine carcinoma, which is strictly defined as over 90% of tumor cells showing apocrine differentiation, is a rare variant of breast cancer. Here we report an uncommon case in which apocrine carcinomas developed concurrently in both breasts; in addition, a sarcomatoid spindle cell lesion was coincident in the right breast. Both apocrine carcinomas were immunohistochemically negative for estrogen receptor (ER) and progesterone receptor (PgR), but diffusely positive for androgen receptor (AR), GCDFP-15, and HER2. The presence of intraductal components in bilateral carcinomas and the absence of lymph node metastasis suggested that they were more likely to be individual primary lesions rather than metastatic disease. The spindle cell lesion showed a relatively well-circumscribed nodule contiguous with the apocrine carcinoma. HER2 oncoprotein overexpression was observed not only in the apocrine carcinoma, but also in the spindle cell lesion. Since the spindle cell component was intimately admixed with apocrine carcinoma and had focal cytokeratin expression, we diagnosed it as metaplastic spindle cell carcinoma, which was originated from the apocrine carcinoma. To our knowledge, this is the first case report of a patient with synchronous bilateral apocrine carcinomas coinciding with metaplastic carcinoma.

## 1. Introduction

The incidence of strictly defined apocrine carcinoma is less than 1% of all breast cancer cases, although focal apocrine differentiation is frequently observed in usual breast cancers. The criteria proposed for the diagnosis of apocrine carcinoma are more than 90% of tumor cells exhibiting apocrine features [1], characterized by large cells with sharply defined borders, abundant eosinophilic granular cytoplasm, and accumulation of secretory granules in the apical cytoplasm, the so-called apocrine snout. Immunohistochemically, apocrine carcinomas tend to be ER- and PgR-negative and AR-positive and to extensively express GCDFP-15 [1–3].

Metaplastic carcinoma is a group of neoplasms characterized by differentiation of the neoplastic epithelium into squamous cells and/or mesenchymal-looking elements [4]. Metaplastic carcinomas with mesenchymal features account for approximately 1% of all invasive carcinomas. Since synchronous bilateral apocrine carcinomas are themselves very uncommon [5], the coincidence with metaplastic carcinomas is extremely rare.

## 2. Case Report

**Figure 1:**
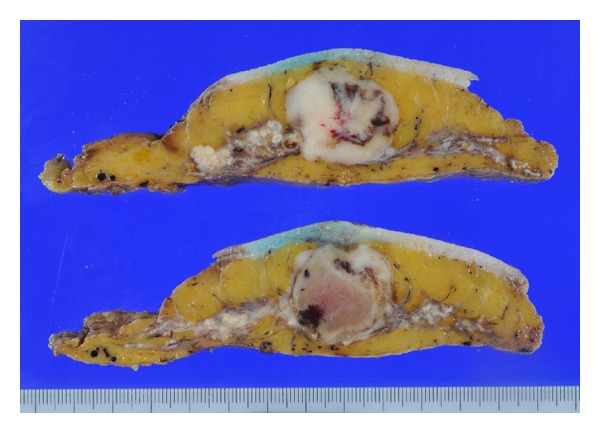
Gross appearance of the right breast tumor. The cut surface showed a relatively well-circumscribed nodule with a whitish tan color, representing metaplastic spindle cell carcinoma. The nodule was contiguous with the apocrine carcinoma component.

A 52-year-old female presented with a right breast lump of 1-month duration. Nipple erosion and bloody discharge were noticed prior to administration. There was no family history of breast or ovarian cancer. Physical examination revealed a firm mass 28 mm in size in the right breast (Figure 1). A mass of 15 mm in the left breast was also disclosed. Preoperative fine-needle aspiration cytology was suggestive of ductal carcinoma. No evidence of distant metastasis was observed by dynamic CT. The patient underwent bilateral total mastectomy and sentinel lymph node resection. Adjuvant chemotherapy treatment was performed after surgery, and the postoperative course was uneventful. To date, there has been no evidence of recurrence two years after surgery.

## 3. Pathological Findings

**Figure 2:**
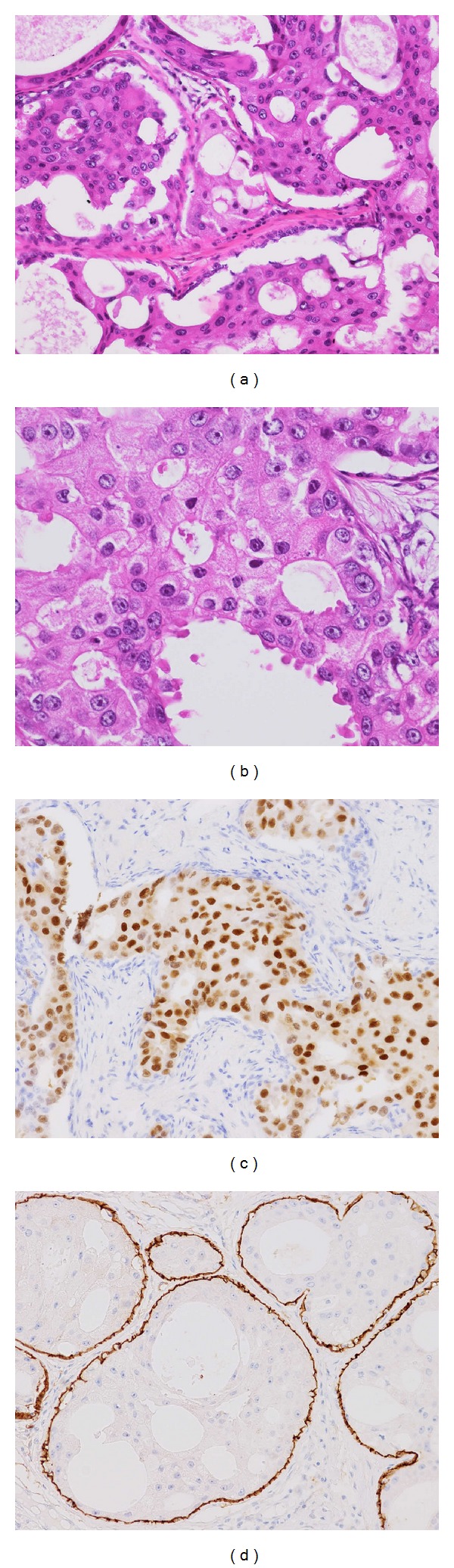
Histological features of apocrine carcinoma in the left breast. ((a) and (b)) HE. The tumor cells had abundant eosinophilic cytoplasm and apocrine snouts. (c) Tumor cells expressed androgen receptors in their nuclei. (d) The intraductal component is represented by a continuous myoepithelial cell layer detected by CD10 staining.

Pathological examination revealed that bilateral breast tumors were invasive carcinoma demonstrating extensive apocrine features. The tumor cells were polygonal in shape with well-defined cell borders and contained abundant eosinophilic cytoplasm. The tumors were composed of compact nests and intraductal components. Apocrine snouts were also observed. Immunohistochemistry demonstrated that the bilateral tumors were consistently negative for ER and PgR, but positive for AR, GCDFP-15, and HER2. These definite apocrine features were observed in over 90% of the lesions in both breasts. Intraductal components showing apocrine features, where a continuous myoepithelial cell layer was confirmed by p63 and CD10 immunostaining, were observed in both breasts and were especially predominant in the left (Figure 2). Comedo-type necrosis was observed in the intraductal lesions. Eczema-like eroded lesion of the right nipple was attributed to intraepidermal spread of the apocrine carcinoma. The apocrine carcinoma extended into the subareolar ducts and demonstrated a Pagetoid appearance. Neither vascular invasion nor metastasis in sentinel nodes was seen.

**Figure 3:**
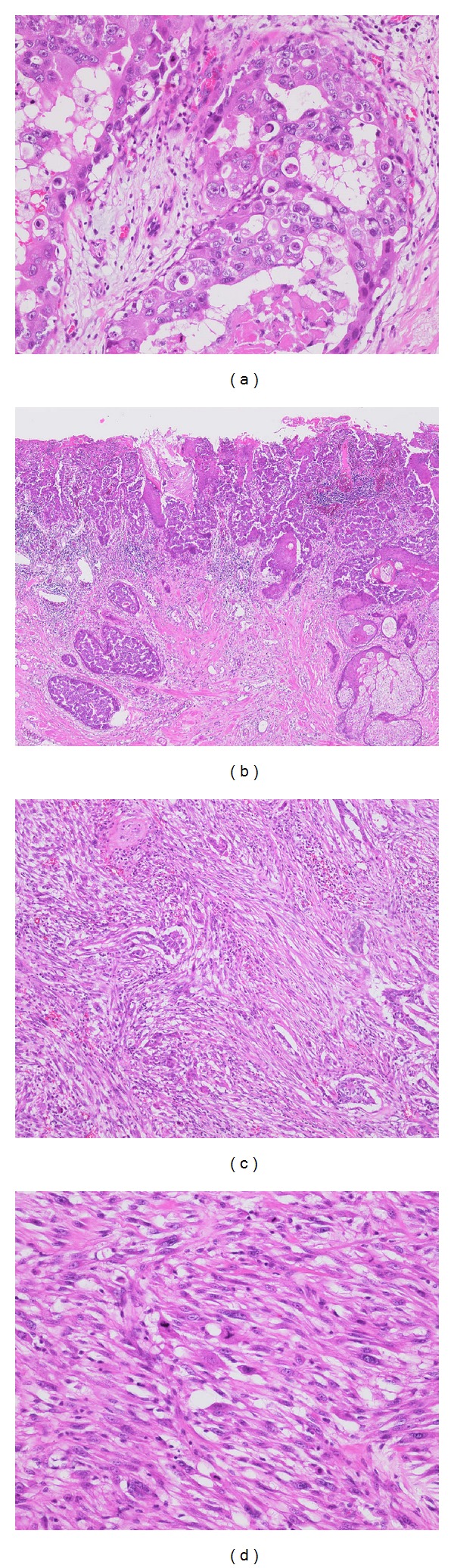
Histological features of apocrine and metaplastic carcinomas in the right breast. (a) HE. Apocrine carcinoma including intraductal components. (b) Intraepithelial invasion in nipple erosion had a Pagetoid appearance. (c) The apocrine carcinoma was admixed with the sarcomatoid spindle cell lesion. (d) The spindle cell lesion showed high nuclear polymorphism and atypia. Atypical mitotic figures are seen.

In addition, the right breast harbored a relatively well-circumscribed firm nodule which was a whitish tan in color and measured 30 mm. The nodule was totally composed of fibroblast-like spindle cells interspersed with varying collagen bundles. The spindle cells were arranged in fascicles, occasionally in storiform pattern, and the nuclei were pleomorphic with coarse chromatin and a significant number of mitotic figures (Figure 3). The spindle cell lesion contained bizarre multinucleated-giant cells and coagulative necrosis. The spindle cell lesion was contiguous with the apocrine carcinoma and a mixture of both components was observed at the periphery. The leaf-like pattern of phyllodes tumors was not observed.

**Figure 4:**
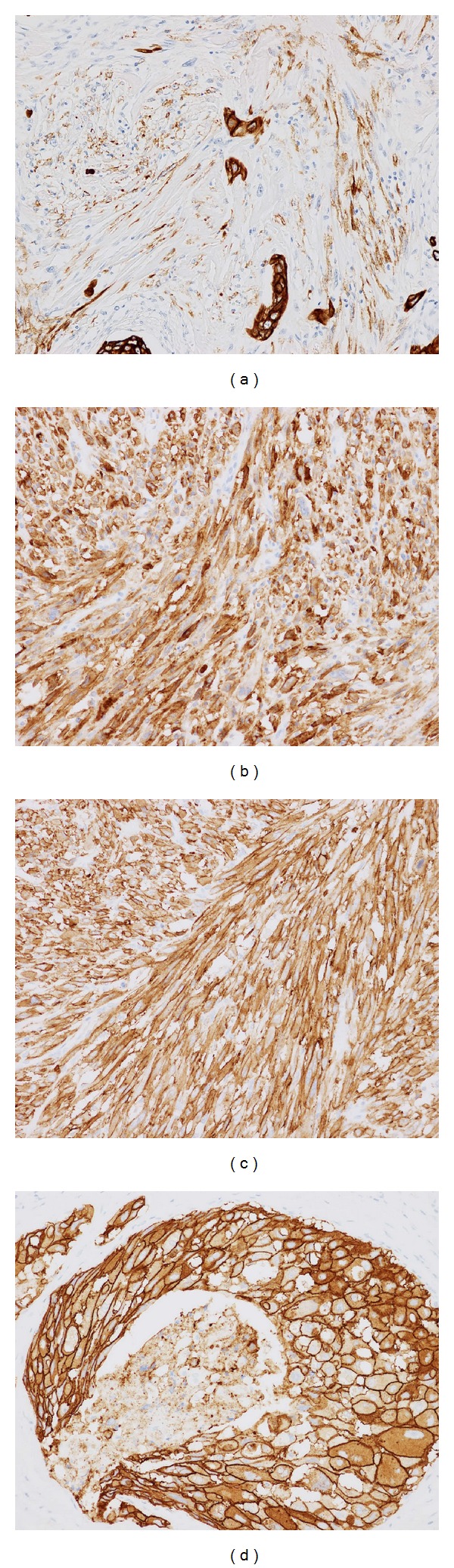
Immunohistochemical staining of the right breast carcinoma. (a) Spindle-shaped tumor cells were focally and weakly positive for cytokeratin CAM5.2, but diffusely and strongly positive for (b) CD10 and (c) HER2 oncoprotein. (d) Apocrine carcinomas strongly expressed HER2 in their cell membrane.

Immunohistochemically, the spindle cell lesion was diffusely positive for vimentin and CD10 and focally positive for alpha-smooth muscle actin. A small number of spindle cells were positive for cytokeratin CAM5.2, whereas most CKs (AE1/AE3, 34*β*E12, and CK5/6) and p63 were negative. The spindle cells were also constantly negative for ER, PgR, and AR. Intriguingly, extensive membranous immunoreactivity for HER2 was observed in the spindle cells as well as the apocrine carcinomas (Figure 4). The immunohistochemical results are listed in Table 1.

## 4. Discussion

The prevalence of synchronous bilateral breast cancer is less than 2% of all breast cancers [6]; however, the definition of synchronous multiple cancers is somewhat unclear in the literature, especially in terms of time interval. In general, a new primary carcinoma diagnosed within 2 months is considered synchronous, whereas 12 months would seem more appropriate from an epidemiological point of view [7–9]. It has been recognized that synchronous bilateral breast tumors most often represent genomically individual primary tumors [10, 11].

It is a debatable issue whether bilateral breast carcinomas are multicentric or metastatic lesions, especially when their histological subtypes are identical. Although different histological subtypes in bilateral breast cancers suggest their multiplicity, it has been documented that synchronous bilateral invasive carcinomas have a high concordance of histological subtypes [12]. It is conceivable that the similarities between the two tumors can be explained by the fact that they develop from the same genetic background and in the same hormonal and environmental status.

The presence of intraductal carcinoma supports the diagnosis of primary breast cancer [13]. In the present case, a significant amount of intraductal components showing apocrine features was observed in both breasts. The existence of intraductal components bilaterally strongly suggested the independent rather than metastatic nature of these lesions. Molecular analysis has demonstrated that synchronous unilateral tumor pairs are often genomically similar, whereas synchronous bilateral tumors are most likely to be nonidentical [10, 14]. However, recent molecular analysis using microarray-based comparative genomic hybridization demonstrated an exceptional case in which even bilateral tumors containing an intraductal component nevertheless seemed most likely to be contralateral tumor spread [10]. Thus, extensive molecular analysis may be needed to conclude whether any two histologically identical breast tumors are intrinsically independent.

Sarcomatoid spindle cell carcinomas can be differentiated from true sarcomas such as malignant phyllodes tumors or periductal stromal sarcomas. It has been proposed that NOS-type sarcoma with CD10 expression arises from stem cells and differentiates to myoepithelial cells to some degree, but the histogenesis remains uncertain [15]. Recent studies have suggested that the immunophenotype with CD10 expression represents a myoepithelial feature, and CD10 is one of the useful markers to track stem cells in breast carcinomas, especially precursors to sarcomatoid metaplastic carcinomas [16]. Since some NOS-type sarcoma with CD10 expression and most metaplastic carcinoma show positivity for *α*-SMA and p63, differential diagnosis can be extremely difficult. Several antibodies are useful in differential diagnosis between metaplastic carcinoma and true sarcoma, although there is no consensus on the minimal antibody panel. The expression of CD34 and Bcl-2, which is generally seen in malignant phyllodes tumors and periductal stromal sarcomas, has not been observed in this case. Moreover, the fact that the spindle cell components were intimately admixed with apocrine carcinoma and were focally positive for CAM5.2 strongly suggested the possibility of metaplastic carcinoma rather than sarcomas [4]. It should be noted that the expression of cytokeratins in metaplastic carcinomas is frequently focal.

It has been reported that metaplastic carcinomas are consistently negative for ER and PgR and generally do not overexpress HER2 [17]; however, the present case maintained extensive HER2 positivity not only in the apocrine carcinoma including the intraductal components but also in the spindle cell lesion. Since the expression of HER2 by the spindle cell component is unusual, the consistent HER2 overexpression in the spindle cell carcinoma strongly suggested its origin in the apocrine carcinoma. Although HER2-positive breast cancers tend to behave more aggressively, high HER2 expression meets the eligibility for anti-HER2 antibody therapy.

In summary, we present a very rare case of HER2-positive metaplastic spindle cell carcinoma coinciding with synchronous bilateral apocrine carcinomas.

## Figures and Tables

**Table 1:** Immunohistochemical results for each tumor morphology.

Immunohistochemistry	Left apocrine	Right apocrine	Spindle
ER	−	−	−
PgR	−	−	−
AR	+	+	−
GCDFP-15	+	+	−
HER2	+	+	+
Cytokeratin CAM5.2	+	+	Focal
Cytokeratin AE1/AE3	+	+	−
Cytokeratin 34*β*E12	Focal	Focal	−
Cytokeratin 5/6	−	−	−
Vimentin	−	−	+
CD10	−	−	+
*α*-Smooth muscle actin	−	−	Focal
h-Caldesmon	−	−	−
Bcl-2	−	−	−
CD34	−	−	−
p63	−	−	−
MIB-1 index	13.0%	10.5%	37.3%

+: positive; −: negative.
